# The inwardly pointing knee angle: A novel computed tomography‐based measurement technique and validation study

**DOI:** 10.1002/jeo2.70938

**Published:** 2026-10-11

**Authors:** Ricardo David Manilov, Angel Sebastián Maldonado, Adrián Bórbore, Estefanía Ivana Vrancovich, Raúl Oscar Cáceres Orellano, Daniela Bravo, Felix Ferner, Matthieu Ollivier

**Affiliations:** ^1^ Orthopedic Surgery Department Clínica Mercedario San Juan Argentina; ^2^ CIMAC San Juan Argentina; ^3^ Orthopedic Surgery Department Sana Klinik Lichtenfels Lichtenfels Germany; ^4^ Department of Orthopaedic Surgery University Medical Center Rostock Rostock Germany; ^5^ Institute of Movement and Locomotion Assistance Publique—Hôpitaux de Marseille (AP‐HM) Marseille France

**Keywords:** anterior knee pain, foot progression angle, internal knee torsion, inwardly pointing knee angle, patellofemoral joint, tomographic protocol, torsional disorder

## Abstract

**Purpose:**

Inwardly pointing knee (IPK) is a clinically recognized manifestation of torsional malalignment in patients with patellofemoral pain and instability. However, current imaging protocols primarily quantify isolated components of malalignment, such as femoral anteversion or tibial torsion, without also capturing the net orientation of the knee relative to the direction of gait. The purposes of this study were to describe a functional computed tomography (CT) protocol for patellofemoral assessment and to introduce and validate the measurement of the inwardly pointing knee angle (IPKA).

**Methods:**

A total of 1230 bilateral CT examinations, performed between 2017 and 2025 in patients with anterior knee pain and/or patellofemoral instability, were retrospectively reviewed. Patients were positioned according to their individual foot progression angle (FPA), documented from plantar footprints obtained during walking before image acquisition. CT scans were obtained with the knees in full extension (0°) and at 30° of flexion while the feet remained fixed on the recorded FPA footprint. In addition to conventional torsional and patellofemoral parameters, IPKA, FPA and AVA were measured on these images. Reliability of IPKA was assessed in 60 randomly selected knees measured by three observers on three occasions, using intraclass correlation coefficients (ICCs), the standard error of measurement (SEM) and the smallest detectable change (SDC).

**Results:**

The mean IPKA was 13.97° ± 7.46° at 0° of flexion and 6.52° ± 5.56° at 30° of flexion. Interobserver reliability was excellent at both 0° (ICC 0.962) and 30° (ICC 0.986) of knee flexion and intra‐observer agreement was excellent (ICC 0.972–0.978 in full extension and 0.989–0.992 at 30°). The SEM was 1.76° in full extension and 0.88° at 30°, with corresponding SDC of 4.88° and 2.43°.

**Conclusions:**

This functional CT protocol enables assessment of the net axial orientation of the knee relative to the body's line of progression during gait, rather than the assessment of isolated torsional variables alone. IPKA demonstrated high reproducibility and may serve as a useful adjunct in the preoperative evaluation of patients with patellofemoral pain or instability and suspected torsional malalignment. IPKA provides a reproducible, objective measure of the combined torsional effect of the acetabulum, femur, tibia and FPA during gait and, pending validation of normative values and clinically meaningful thresholds, may serve as an adjunct to conventional torsional imaging in the functional assessment of complex patellofemoral disorders.

**Level of Evidence:**

Level III, diagnostic cross‐sectional study.

**Abbreviations**
AVA: acetabular version angle
CT: computed tomography
FPA: foot progression angle
ICC: intraclass correlation coefficient
IPK: inwardly pointing knee
IPKA: inwardly pointing knee angle
MRI: magnetic resonance imaging
SDC: smallest detectable change
SEM: standard error of measurement
TT–TG: tibial tuberosity–trochlear groove

## INTRODUCTION

Patellofemoral malalignment is associated with anterior knee pain, instability and progressive chondral degeneration. Its aetiology is multifactorial and appropriate treatment depends on identifying the dominant factors contributing to abnormal patellofemoral mechanics. In contrast to the femorotibial joint, which mainly transmits load along the longitudinal axis of the limb, the patellofemoral joint is highly sensitive to alterations in axial alignment, because these changes influence contact area, joint reaction force and shear stress [3, 6, 7, 15].

Femoral and tibial torsional variations are well‐established contributors to patellofemoral malalignment. Increased femoral anteversion and external tibial torsion may produce compensatory internal orientation of the knee within the lower‐limb axis, often resulting in the clinical appearance of a ‘squinting patella’ or inwardly pointing knee (IPK) [7, 9, 12, 15, 17]. Cooke et al. first described IPK as an underrecognized source of treatment failure, and subsequent authors have refined the syndrome and emphasized the importance of torsional analysis [1, 8]. Correction of torsional malalignment is also indicated for reasons other than patellofemoral pain, including in‐toeing or out‐toeing gait disturbance, femoroacetabular impingement and compensatory deformity at adjacent joints; the present protocol and IPKA measurement are relevant to this broader population, even though the validation cohort was drawn from patients with anterior knee pain and/or instability. Recent reviews of the evaluation and treatment of lateral patellar instability similarly emphasize rotational malalignment as a central, modifiable contributor among its multifactorial causes [21].

Most current imaging protocols quantify the individual components of torsional deformity—femoral anteversion, tibial torsion and acetabular version—and this remains essential for localizing a deformity and planning surgical correction. These component measurements do not, however, by themselves capture the combined functional result perceived clinically: the orientation of the knee relative to the line of body progression during gait. In practice, patellofemoral symptoms are determined not by femoral anteversion or tibial torsion in isolation, but by the cumulative effect of the acetabulum, femur, tibia, knee and foot progression angle (FPA). This combined effect determines the net rotational orientation of the knee during walking. Isolated femoral or tibial torsion is not, by itself, necessarily symptomatic; the resulting patellofemoral shear force and reduction in joint contact area depend on how that torsion combines with the individual's FPA. For example, increased external tibial torsion combined with a correspondingly increased (out‐toed) FPA may leave the knee oriented essentially forward, whereas the same tibial torsion combined with a neutral FPA may produce a clinically apparent IPK (Figure 1) [17].

**Figure 1:**
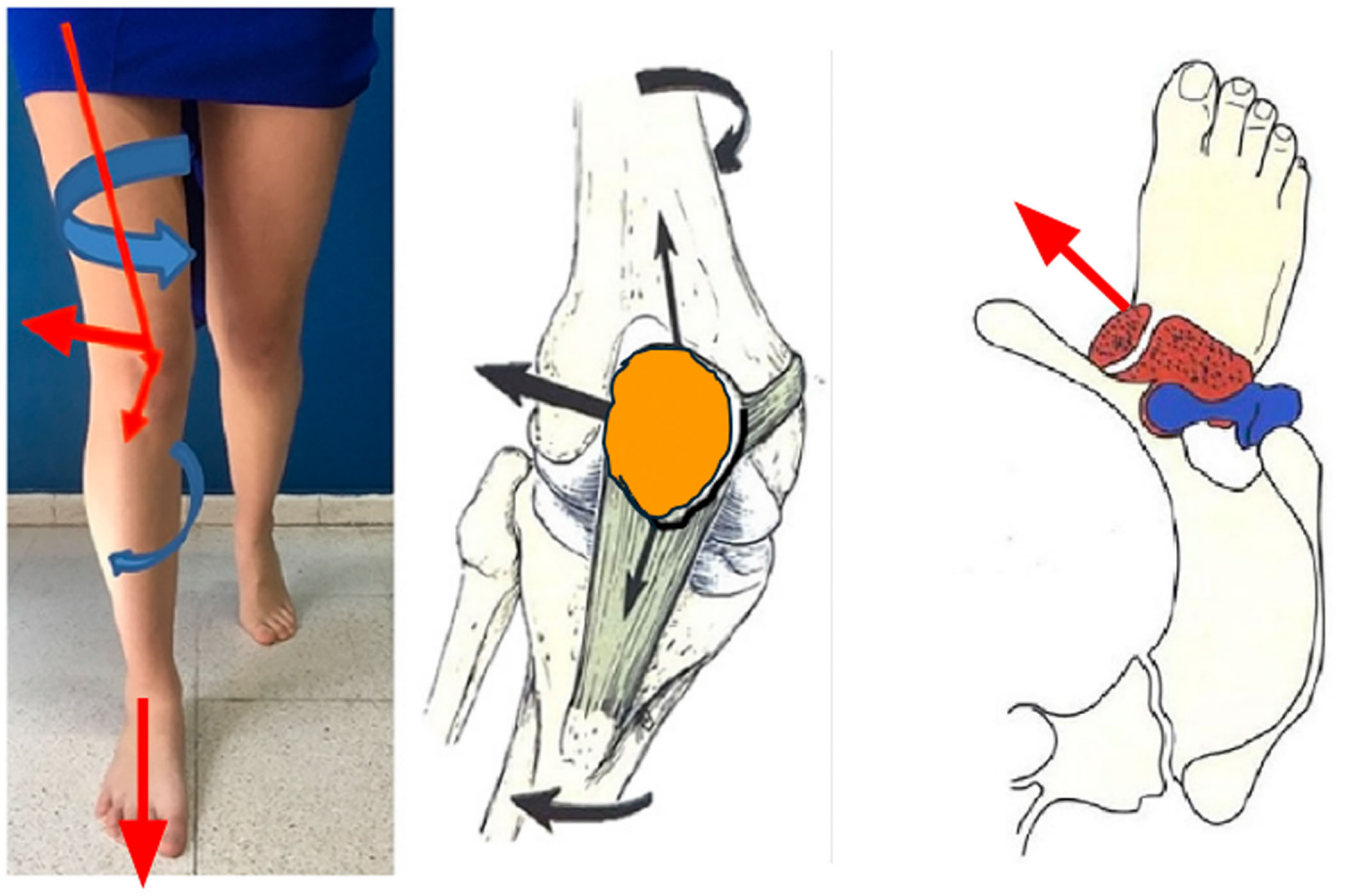
(a) Photograph of a patient taking a step, with the foot in the walking position. Femoral internal rotation is observed (blue curved arrow, right), resulting in an increased Q angle and a lateral force vector (proximal red arrows). There is relative external tibial torsion compared with the internally rotated position of the knee relative to the axis of the right lower limb (IPK). The foot position (FPA) during walking generates compensatory internal knee torsion. (b) Pathophysiological diagram illustrating how internal femoral torsion and external tibial torsion increase the *Q* angle, producing lateral force vectors that affect the patellofemoral joint. (c) Diagram showing the overlap of the pelvis, hip, knee and foot, demonstrating how foot positioning in a walking stance generates internal knee torsion along its axis (IPK, red arrow), creating a force imbalance that must be quantified to plan correction.

Yet despite the significant influence of IPK on the patellofemoral joint, no described imaging method was identified that directly measures the net inward orientation of the knee relative to gait progression. The purpose of this study was therefore to develop a simple, reproducible tomographic method for measuring the inwardly pointing knee angle (IPKA) within a comprehensive and complete functional patellofemoral computed tomography (CT) protocol and to evaluate the reliability of this measurement. It was hypothesized that IPKA could be measured with excellent intra‐ and inter‐observer reliability at both 0° and 30° of knee flexion.

## MATERIALS AND METHODS

### Study population

A database of bilateral CT examinations, obtained between 2017 and 2025 in patients presenting with anterior knee pain and/or patellofemoral instability, was retrospectively reviewed. All patients were evaluated bilaterally. Exclusion criteria were open physis, previous knee surgery and tricompartmental arthritis. Approved by the Ethics Committee (CEI) of the Universidad Católica de Cuyo, San Juan, Argentina; approval number 122.M2.2026‐018.

### Imaging protocol

The torsional measurement was performed for all patients following a standardized protocol. Before CT acquisition, each patient walked across paper placed on the floor to document the plantar footprints and the individual FPA during gait. The floor was lightly dampened with medical‐grade alcohol so that the footprints transferred to the paper; the outlines were then traced with pencil before evaporation (Figure 2). These footprints were subsequently used as the positioning template during CT acquisition.

**Figure 2:**
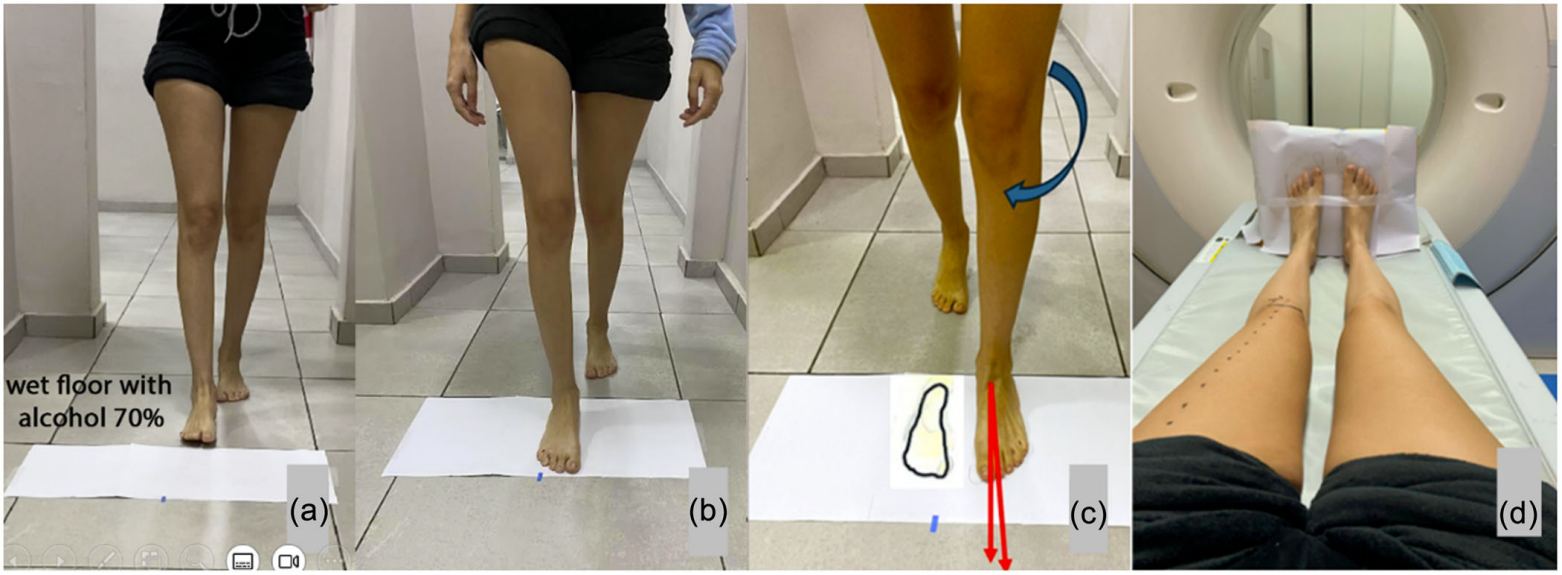
Footprint acquisition for patient positioning in a functional posture, simulating gait. (a) The patient walks and wets the foot with 70% alcohol previously applied to the floor. (b) The patient records the footprint and FPA on paper secured to the floor. (c) The technician outlines the footprint with a pen before it dries. (d) The patient is then positioned over the FPA footprints, reproducing the posture adopted during walking. FPA, foot progression angle.

All scans were obtained using a 16‐slice multislice CT scanner (General Electric BrightSpeed Elite; software version 17BW11.2_SP1‐1‐1.HP_S_P16_G_HLT). Axial images were acquired at 1.3‐mm slice thickness, 120 kV, 49 mA, matrix 512 × 512 and field of view 43 × 43 cm. Patients were positioned supine on the CT table with the feet toward the gantry. The feet were secured over the previously recorded FPA footprint so that the lower limbs reproduced the patient‐specific walking position (Figure 3). Images were acquired with the knees in full extension and at 30° of flexion while the quadriceps remained relaxed and the feet were kept fixed with adhesive tape. Knee flexion was verified with a goniometer. This method simulates the internal knee torsion that the knee adopts in space relative to the imagined line of walking progression. It should be noted that this protocol retains the conventional static measurements of femoral anteversion and tibial torsion obtained as in traditional protocols, and adds IPKA, FPA and acetabular version angle (AVA). These additional parameters are acquired with the patient supine (not weight‐bearing) and the foot fixed on the individually recorded FPA footprint, so as to simulate the rotational alignment of the limb adopted during gait; this simulated‐gait positioning does not reproduce hip or ankle joint motion.

**Figure 3:**
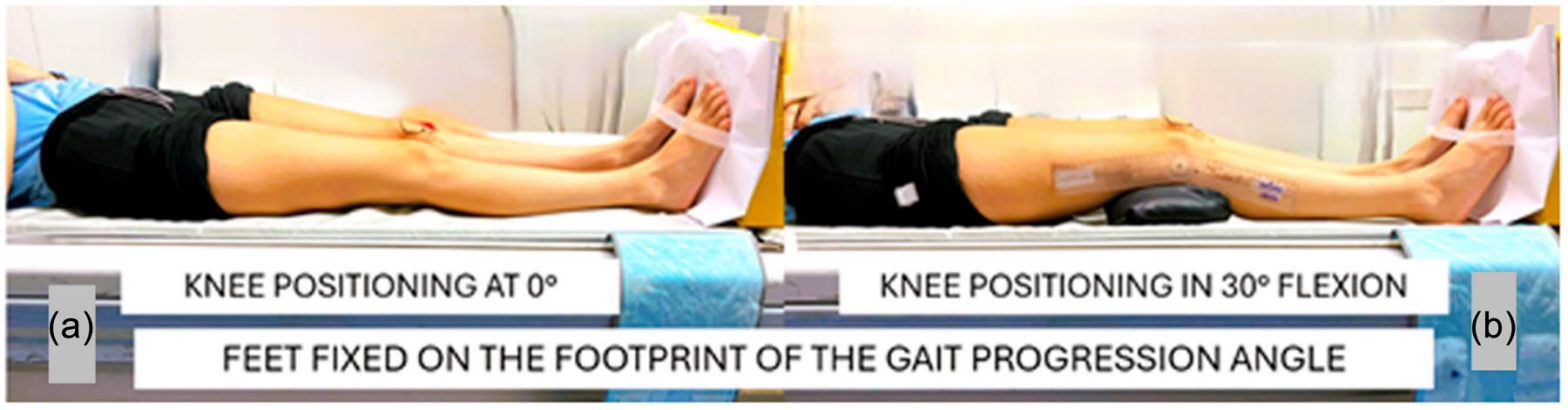
Standardized patient positioning for inwardly pointing knee (IPK) assessment. The feet are secured with tape at 0° (a) and 30° (b) of knee flexion. Throughout the examination, the feet remain fixed over the documented foot progression angle (FPA) footprint to ensure consistent alignment and reproducibility.

At 0° of knee flexion, image acquisition included the hip (from the anterior superior iliac spine to the lesser trochanter), the knee (from two slices proximal to the superior pole of the patella to two slices distal to the tibial tuberosity) and the ankle/foot (from the distal tibiofibular syndesmosis through the entire foot). At 30° of knee flexion, images were acquired from two slices proximal to the superior pole of the patella to two slices distal to the tibial tuberosity.

### Measurements

In addition to the standard patellofemoral measurements—including femoral anteversion and tibial torsion—IPKA, FPA and the AVA were assessed; the latter was measured according to the Tönnis and Heinecke method [18]. IPKA was measured at both 0° and 30° of knee flexion while the feet remained fixed on the individualized FPA footprint (Figure 4). On the axial image, the femoral bicondylar line was drawn and its angle relative to a horizontal reference line parallel to the floor was measured; this horizontal reference line was perpendicular to the presumed line of body progression during gait. Positive values indicated internal rotation of the entire knee relative to the direction of progression (IPK), whereas negative values indicated external rotation. IPKA is typically greater at 0° than at 30° of flexion [9, 10]. Although IPKA is measured using the femoral bicondylar axis as its anatomical landmark, because this axis is captured with the limb positioned according to the patient's individual FPA, its inclination represents the net resultant of the proximal (acetabular, femoral) and distal (tibial, foot) contributions that together determine the knee's orientation during gait—rather than an isolated, purely femoral measurement.

**Figure 4:**
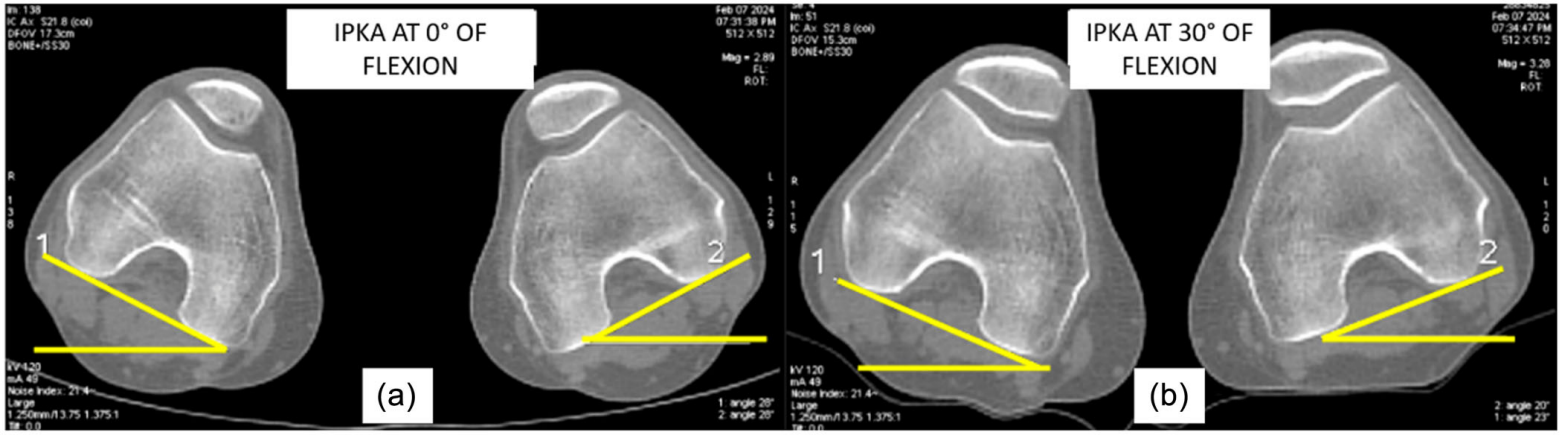
Inwardly pointing knee (IPK) angle measurement technique at 0° (a) and 30° (b). The bicondylar line is drawn, and the angle is determined at its intersection with a horizontal reference line parallel to the floor, which is perpendicular to the estimated direction of body progression during gait. Positive values represent internal knee torsion (convergent alignment); negative values indicate external knee torsion (divergent alignment) relative to the axis of progression. IPKA, inwardly pointing knee angle.

The FPA quantifies the degree of external rotation of the foot relative to a reference axis representing the direction of body progression during simulated gait. FPA was measured as the angle between the longitudinal axis of the foot and a line perpendicular to the floor (Figure 5). The longitudinal axis of the foot was drawn from the centre of the calcaneus to the second metatarsal. Positive values represented out‐toeing and negative values represented in‐toeing. During imaging, the feet remained fixed on the recorded footprint to preserve this functional position. The reference range is 5°–15° [14, 16].

**Figure 5:**
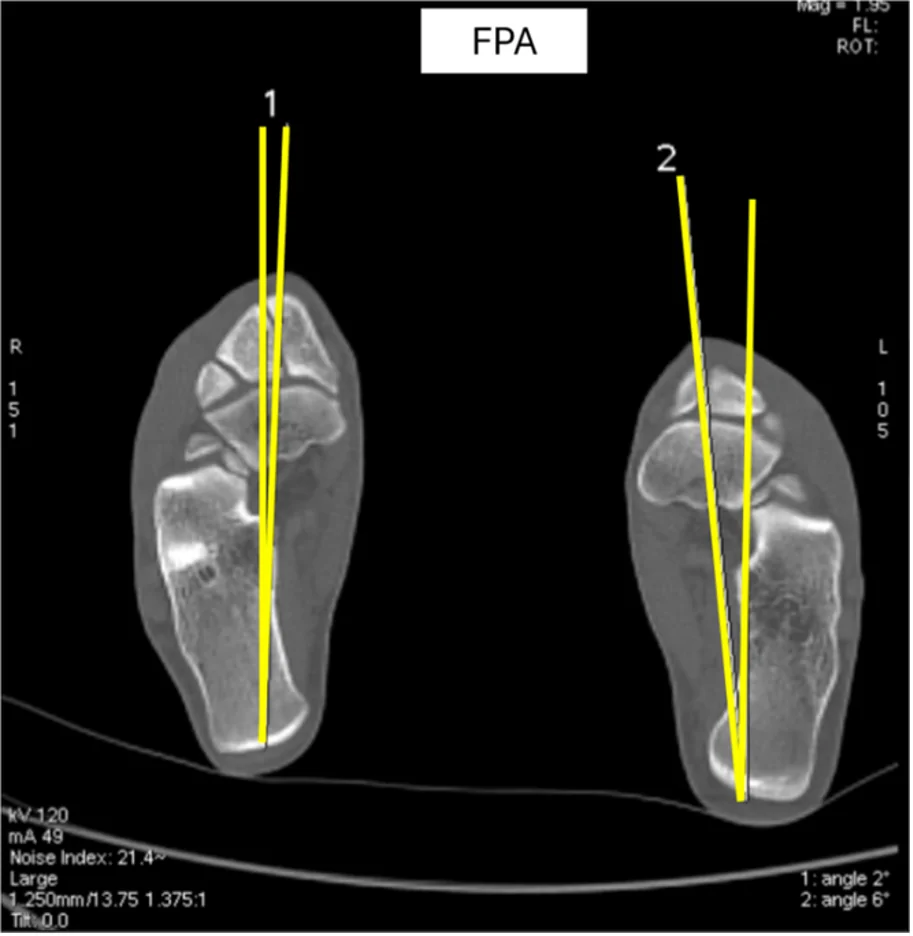
Foot progression angle (FPA) measurement. The angle is formed by the intersection of two lines: one from the centre of the calcaneus to the centre of the second metatarsal and the other from the centre of the calcaneus perpendicular to the horizontal line (the imaginary line of body direction while walking).

AVA was measured on the axial slice with the widest acetabulum as the angle between a line perpendicular to the horizontal and a line connecting the anterior and posterior acetabular walls (Figure 6) [18].

**Figure 6:**
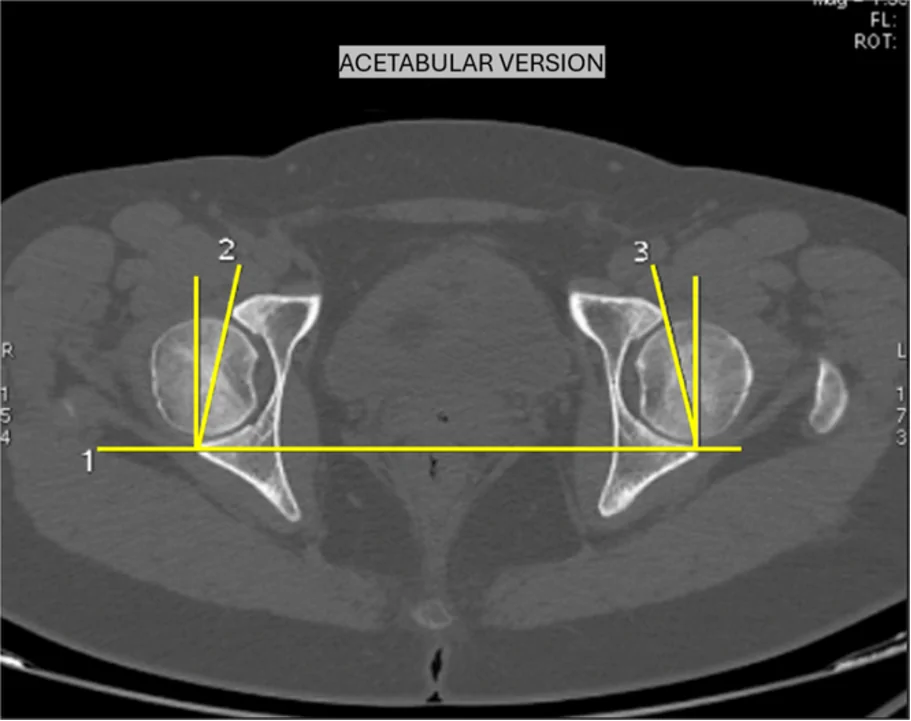
Acetabular version angle (AVA) measurement, defined by the intersection of two lines: one connecting the anterior and posterior acetabular wall edges and the other sagittal to the pelvic plane (perpendicular to the horizontal line).

Additional measurements obtained using the protocol included conventional patellofemoral and torsional parameters. These variables comprised: sulcus angle, patellar tilt angle, trochlear inclination angle, sulcus–tuberosity distance, tibial tubercle lateralization, sulcus medialization, femorotibial rotation, femoral anteversion, external tibial torsion and external tibial torsion measured relative to the epicondylar line.

### Statistical and reliability analysis

To evaluate both intra‐ and inter‐observer reliability of IPKA, 60 knees were assessed under two conditions: full extension (0°) and 30° of knee flexion. Each knee was independently measured by three observers, with three repeated measurements per observer at 1‐week intervals to minimize recall bias and ensure measurement independence. Reliability was assessed using intraclass correlation coefficients (ICCs), applying two‐way mixed‐effects models with absolute agreement for intra‐observer reliability (ICC 3,1) and two‐way random‐effects models for inter‐observer reliability (ICC 2,1), based on the mean of repeated measurements. Measurement error was quantified using the standard error of measurement (SEM) and the smallest detectable change (SDC). To evaluate potential systematic differences, linear mixed‐effects models were applied, including rater, repetition (week) and knee position as fixed effects and knee as a random effect. Analyses were also performed separately for each knee position to assess condition‐specific reliability. ICC values were interpreted according to established guidelines: below 0.40 poor, 0.40–0.59 fair, 0.60–0.74 good and above 0.75 excellent. Statistical significance was set at *p* < 0.05. All analyses were conducted using RStudio (RStudio Team).

## RESULTS

A total of 615 patients (1230 knees) were included. The cohort was approximately 80% female, with a mean age of 25.4 years (range, 15–52 years). Cohort characteristics are summarized in Table 1.

**Table 1:** Study cohort demographics.

Characteristic	Value
Patients, *n*	615
Knees, *n*	1230
Female, %	79.9
Male, %	20.1
Mean age, years (range)	25.4 (15–52)

### IPKA descriptive values

Across all measured knees, the mean (± standard deviation) IPKA was 13.97° ± 7.46° at 0° of flexion (*n* = 1247) and 6.52° ± 5.56° at 30° of flexion (*n* = 1263), consistent with the expected decrease in net internal knee torsion as the knee flexes (Table 2). In an exploratory descriptive analysis, IPKA at full extension was greater in women than in men (approximately 15° vs. 10°) and was symmetric between sides, in keeping with the higher prevalence of internal knee torsion previously reported in women.

**Table 2:** Descriptive statistics for the IPKA at 0° and 30° of knee flexion.

Parameter	IPKA 0° (full extension)	IPKA 30° (flexion)
Number of knees	1247	1263
Median (°)	14.00	6.00
Mean (°)	13.97	6.52
Standard deviation (°)	7.46	5.56
Standard error (°)	0.21	0.16
95% CI (°)	13.56–14.39	6.21–6.82

Abbreviations: CI, confidence interval; IPKA, inwardly pointing knee angle.

### Reliability and measurement error

At full knee extension (0°), intra‐observer reliability was excellent across all raters (ICC 0.972–0.978), and inter‐observer reliability was also excellent (ICC 0.962). At 30° of flexion, intra‐observer reliability was excellent (ICC 0.989–0.992) and inter‐observer reliability was excellent (ICC 0.986). Measurement‐error analysis showed a SEM of 1.76° and an SDC of 4.88° in full extension, and a SEM of 0.88° and an SDC of 2.43° at 30° of flexion (Table 3; Figure 7). The linear mixed‐effects models revealed no clinically relevant systematic effect of rater or repetition, supporting the robustness of the measurement across observers and occasions.

**Table 3:** Reliability and measurement error of IPKA.

Parameter	0° (full extension)	30° (flexion)
ICC, inter‐observer	0.962	0.986
ICC, intra‐observer	0.972–0.978	0.989–0.992
SEM (°)	1.76	0.88
SDC (°)	4.88	2.43

Abbreviations: ICC, intraclass correlation coefficient; IPKA, inwardly pointing knee angle; SDC, smallest detectable change; SEM, standard error of measurement.

**Figure 7:**
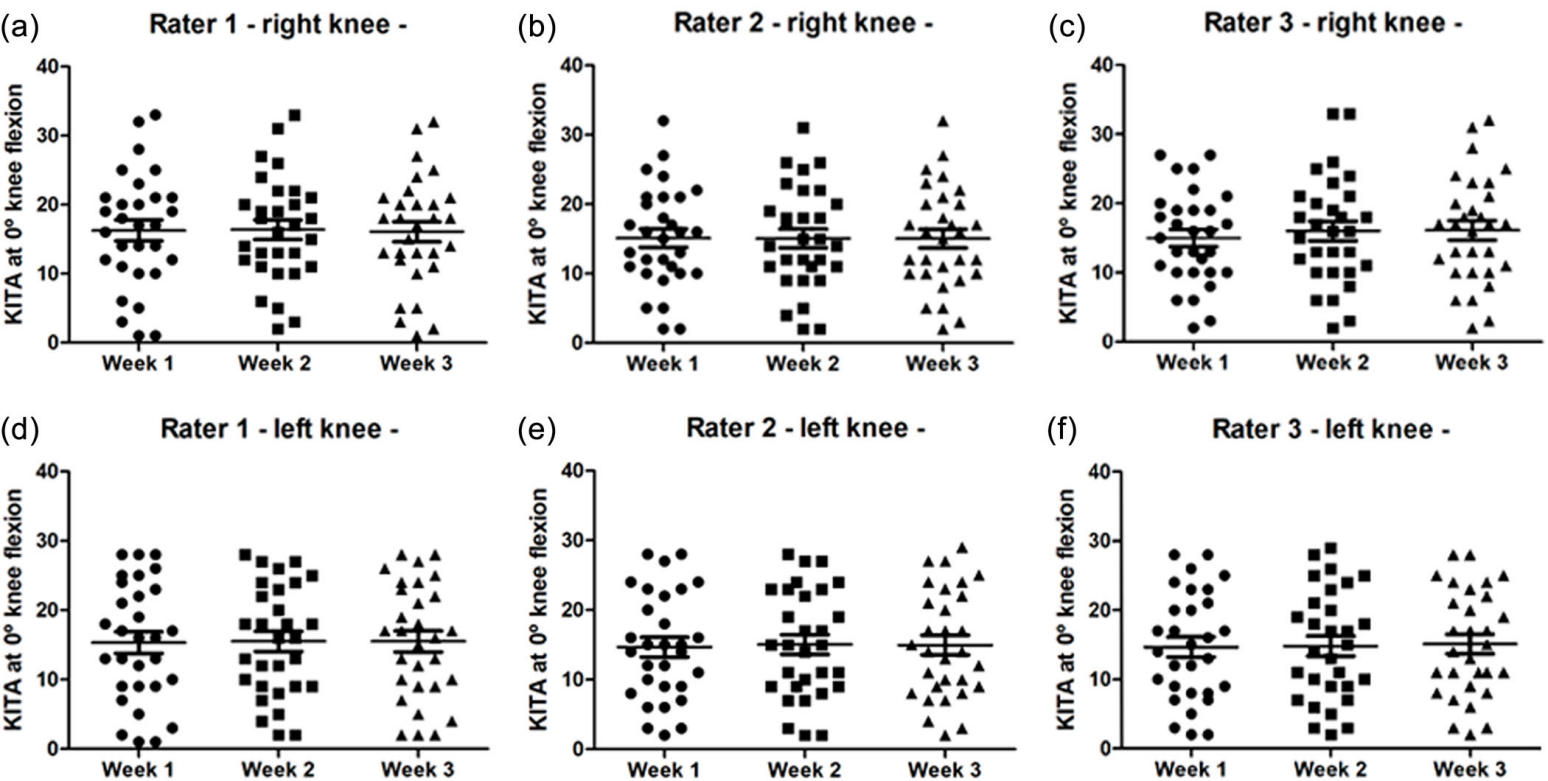
Observer variability and validation of the IPK angle. Each observer performed three measurements of 30 patients (60 knees) at 1‐week intervals. Reliability reflects not only the degree of correlation but also the agreement between measurements; intraclass correlation coefficients (ICCs) were used as the reliability index in test–retest, intra‐rater and inter‐rater analyses. (a) Rater 1—right knee. (b) Rater 2—right knee. (c) Rater 3—right knee. (d) Rater 1—left knee. (e) Rater 2—left knee. (f) Rater 3—left knee.

## DISCUSSION

The most important finding of this study is that IPKA can be measured reproducibly with a functional CT protocol that positions the patient according to the individual FPA recorded during walking. This protocol shifts the focus from isolated torsional measurements to the net spatial orientation of the knee relative to the body's line of progression, which may better reflect the clinical appearance of IPK and thus the mechanical environment of the patellofemoral joint during gait.

Current torsional workups commonly report femoral anteversion, tibial torsion and tibial tuberosity–trochlear groove (TT–TG) distance, but these variables do not fully capture the final position of the knee during gait. Two patients with similar femoral or tibial torsional values may have different clinical presentations because the eventual orientation of the knee is also influenced by AVA and FPA. As a result, the functional position of the knee—and the resultant patellofemoral contact area and joint stresses—may not be accurately captured by isolated measurements. By directly measuring IPKA, the proposed protocol quantifies this combined effect, equipping the surgeon with a method for assessing the functional position of the knee during gait, which is fundamental for identifying the torsional disorders ultimately responsible for reduced articular contact area and increased shear stress.

Fundamentally, this protocol introduces a method to systematically quantify the clinical observation of IPK, which is increasingly recognized as a key determinant of patellofemoral symptoms [1, 17]. Cooke et al. first highlighted IPK as an unrecognized cause of treatment failure in patients with increased external tibial rotation, in whom this factor was not initially corrected and who required subsequent tibial derotational osteotomy [1]. Teitge further emphasized the role of rotational malalignment in patellofemoral pain and instability [17]. More recently, Liße et al. refined the concept of the IPK syndrome, demonstrating excellent results treating these patients with double‐level derotational osteotomies [8]. The present study builds on these works by providing a method to systematically quantify this condition using a functionally positioned CT protocol.

The literature does not currently establish the normal (physiological) values of internal knee torsion and IPK. According to the pathophysiology described by Teitge, IPK is ultimately responsible for the destabilizing forces that generate symptoms [17]. Alterations in femoral or tibial torsion are not, by themselves, necessarily pathological; only when increased femoral anteversion and/or external tibial torsion combine with a given FPA and an AVA that cannot compensate through internal hip rotation does the knee experience excessive internal torsion. This, in turn, leads to an imbalance in joint reaction forces and destabilization of the patella within the trochlea. This underscores the potential value of measuring IPKA as an adjunct to conventional torsional assessment, pending confirmation of normative values and outcome‐based thresholds in future studies.

Studies using the same protocol with magnetic resonance imaging (MRI) are ongoing and will enable assessment of IPKA in asymptomatic individuals without radiation exposure, establishing an asymptomatic comparison group and the pathologic threshold for IPKA. Clinical experience at our institution indicates that individuals who do not improve with conservative management and require derotational osteotomy—tibial, femoral or double‐level—demonstrate IPKA in extension exceeding 10°, leading us to hypothesize that the physiological tolerance threshold approximates this value. This observation is retrospective and hypothesis‐generating only: it has not been validated using asymptomatic controls, diagnostic‐accuracy analyses, clinical correlations or treatment outcomes, and must not presently be used to guide diagnosis or treatment selection. Future prospective studies are necessary to substantiate or refute it.

We consider IPKA for the patellofemoral joint conceptually analogous to joint line obliquity (JLO) for the femorotibial joint: a composite, functionally meaningful angle that summarizes the net effect of multiple proximal and distal contributors, rather than an isolated structural measurement. Our surgical goal in derotational osteotomy is to realign the knee with the body's direction of progression during walking, rather than to normalize femoral or tibial torsion values in isolation [20]. We emphasize that no IPKA threshold—including the approximately 10° value discussed above—has been validated using asymptomatic controls, diagnostic‐accuracy analyses, clinical correlations or treatment outcomes; it therefore cannot presently support diagnostic or treatment‐selection decisions and is reported solely as a hypothesis for future prospective study.

We hypothesize that femoral or tibial torsion alone does not generate patellofemoral symptoms; rather, symptoms arise when torsion combines with the individual's FPA in a way that increases patellofemoral shear force or reduces joint contact area. For example, a patient with increased external tibial torsion but a correspondingly increased (out‐toed) FPA may walk with the knee oriented essentially forward, because the distal rotational excess is functionally absorbed by the foot; in our clinical experience, FPA does not appreciably change after derotational osteotomy, suggesting that surgical correction should target the knee's net orientation (IPKA) rather than the isolated long‐bone torsion value. This hypothesis requires prospective validation and is not established by the present reliability data.

With respect to implementation, IPKA demonstrated excellent intra‐ and inter‐observer reliability at both 0° and 30° of knee flexion, supporting its practical use within this functional CT protocol. SEM and SDC were slightly lower at 30°, suggesting marginally less measurement error at this position; because the differences were small and reproducibility was excellent at both positions, this finding is best interpreted as incremental. Notably, the ICCs observed for IPKA compare favourably with published reliability data for established patellofemoral and torsional measurements: in a recent systematic review, TT–TG demonstrated intra‐ and inter‐observer ICCs ranging from 0.74 to 0.99 and 0.71 to 0.98, respectively, while CT‐based femoral anteversion and tibial torsion measurements have shown intra‐observer ICCs of approximately 0.91–0.92 and inter‐observer ICCs of approximately 0.90–0.91. Overall, IPKA is a reproducible and readily applicable measure that can be assessed reliably at either knee position, with performance similar or superior to traditional measures.

The protocol also allows the examiner to interpret conventional measurements in a more comprehensive context. For example, TT–TG distance may be influenced by tibial tubercle lateralization, trochlear groove medialization and femorotibial rotation. Tensho et al. [16] studied these three components and found that femorotibial rotation had the greatest impact on TT–TG distance; however, their analysis could not determine whether the origin was femoral, tibial or both. Tibial tubercle torsion measured by CT has likewise been shown to correlate with patellar height, further illustrating how individual torsional components interact with other patellofemoral parameters [4]. The present protocol assesses the same factors and additionally allows the clinician to contextualize the origin of a rotational abnormality: static femoral anteversion and tibial torsion values localize the abnormality to the femur, tibia or both, while IPKA independently quantifies how that abnormality—combined with FPA and AVA—resolves into the net functional orientation of the knee during simulated gait. Likewise, whereas patellar tilt is traditionally attributed to a tight lateral retinaculum or the absence of medial restraint, in certain patients it may in fact reflect underlying torsional malalignment when the patient is positioned functionally. The value of the protocol therefore lies not only in the measurement of IPKA itself but also in the more integrated interpretation of established patellofemoral parameters.

Further studies are needed to assess the relationship between femorotibial rotation and IPKA. We hypothesize that femorotibial rotation may be influenced by capsuloligamentous laxity and joint‐surface geometry rather than by long‐bone torsion alone [5], consistent with recent evidence linking increased tibiofemoral rotation to anatomical risk factors for lateral patellar instability [19]; however, pre‐ and post‐osteotomy data on femorotibial rotation are currently lacking, and this hypothesis requires prospective validation before any clinical inference is drawn.

Most existing protocols position patients in a comfortable, non‐functional manner, without accounting for the rotational alignment of the knee along its axial axis during daily activities such as walking or ascending and descending stairs [2, 13]. Failure to position the patient in a manner that simulates functional activity can lead to misinterpretation of the torsional forces acting on the knee. During activities such as walking or stair climbing, the foot is naturally aligned according to the individual's FPA, which may result in an IPK that remains undetected if the patient is not positioned in the scanner based on their unique functional FPA footprint (Figure 8). In addition, traditional patellofemoral measurements may be influenced and limited by physiologic variation in femorotibial rotation related to scanner positioning, and there is growing recognition of the need for standardized positioning during patellofemoral assessment. For example, Pascual‐Leone et al. [11] demonstrated that TT–TG values on repeated MRIs in the same patients, obtained a mean of 5 months apart, varied by as much as 80% (from a 21.2% decrease to a 61.1% increase). The protocol described here therefore not only provides a more functional assessment of patellofemoral alignment but may also improve the reproducibility of traditional patellofemoral parameters.

**Figure 8:**
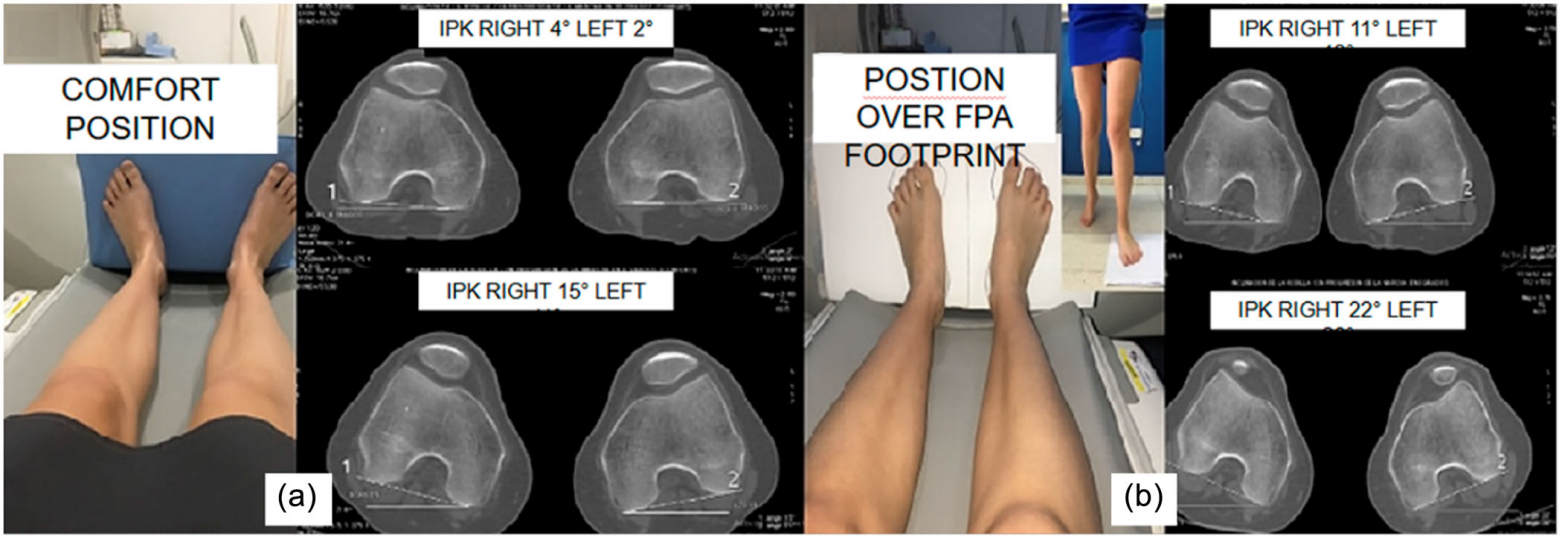
Comparative analysis of IPK measurements in comfort versus functional FPA positions. (a) Patient positioned for CT in the comfort position; IPK at 30° of knee flexion was 4° (right) and 2° (left) and at 0° was 15° (right) and 11° (left). (b) The same patient positioned according to the recorded functional FPA footprint; IPK at 30° was 11° (right) and 12° (left) and at 0° was 22° (right) and 20° (left). The comparison highlights the marked differences between comfort and functional positions, emphasizing that functional assessment more accurately represents the true spatial orientation of the knee. CT, computed tomography; FPA, foot progression angle; IPK, inwardly pointing knee.

### Limitations

First, the study was retrospective; however, the data were collected prospectively and no prognostication was performed, so this is a minor limitation. Second, no asymptomatic control group could be included; because the deformity is often bilateral, the contralateral knee was unsuitable as a control, given the uncertainty regarding potential future symptoms. Finally, the analysis focused on the description and validation of the measurement technique rather than on correlation with symptoms, treatment selection or postoperative outcomes. Because the cohort comprised exclusively symptomatic patients, the present data establish the reproducibility of IPKA but cannot determine its normal physiological range or a clinically meaningful pathological threshold. Consequently, IPKA cannot presently distinguish normal anatomic variation from clinically significant malalignment, and it should not be used in isolation for diagnosis or surgical decision‐making. This limitation will be addressed by the planned MRI‐based study of asymptomatic individuals described above.

## CONCLUSION

This study presents a novel, comprehensive and complete functional CT protocol for patellofemoral assessment that, in addition to conventional intrinsic and extrinsic torsional and patellofemoral variables, includes measurement of the IPKA. IPKA quantifies the net rotational orientation of the knee relative to the body's line of progression—the fundamental driver of lateralizing patellofemoral forces during gait—integrating the combined effects of acetabular version, femoral anteversion, tibial torsion and FPA. The measurement demonstrated high reliability and, pending validation of normative values and clinically meaningful thresholds, may serve as a useful adjunct to conventional torsional imaging in the preoperative evaluation of patients with patellofemoral pain or instability and suspected torsional malalignment. Further research is required to define these normal values and thresholds before IPKA can be used independently in clinical decision‐making.

## AUTHOR CONTRIBUTIONS

Ricardo David Manilov, Angel Sebastián Maldonado, Adrián Bórbore, Estefanía Ivana Vrancovich, Raúl Oscar Cáceres Orellano and Daniela Bravo contributed to study design and to the acquisition, analysis and interpretation of data. Ricardo David Manilov, Felix Ferner, NV and Matthieu Ollivier contributed to drafting the manuscript and revising it critically for important intellectual content. All authors approved the submitted and final versions.

## FUNDING INFORMATION

The authors have no funding to report.

## CONFLICT OF INTEREST STATEMENT

The authors declare no conflicts of interest.

## ETHICS STATEMENT

This study was approved by the Ethics Committee (CEI) of the Universidad Católica de Cuyo, San Juan, Argentina; approval number 122.M2.2026‐018. Written informed consent was obtained from all participants.

## Data Availability

The data that support the findings of this study are available from the corresponding author upon reasonable request.
